# Solution-phase automated synthesis of an α-amino aldehyde as a versatile intermediate

**DOI:** 10.3762/bjoc.13.13

**Published:** 2017-01-17

**Authors:** Hisashi Masui, Sae Yosugi, Shinichiro Fuse, Takashi Takahashi

**Affiliations:** 1 Yokohama University of Pharmacy, 601 Matano-cho, Totsuka-ku, Yokohama 245-0066, Japan,; 2 Laboratory for Chemistry and Life Science, Institute of Innovative Research, Tokyo Institute of Technology, 4259 Nagatsuta-cho, Midori-ku, Yokohama 226-8503, Japan

**Keywords:** acetal formation, amino acid, automated synthesis, Garner’s aldehyde, reduction

## Abstract

A solution-phase automated synthesis of the versatile synthetic intermediate, Garner’s aldehyde, was demonstrated. *tert*-Butoxycarbonyl (Boc) protection, acetal formation, and reduction of the ester to the corresponding aldehyde were performed utilizing our originally developed automated synthesizer, ChemKonzert. The developed procedure was also useful for the synthesis of Garner’s aldehyde analogues possessing fluorenylmethyloxycarbonyl (Fmoc) or benzyloxycarbonyl (Cbz) protection.

## Introduction

Automated synthesis has attracted a great deal of attention in recent years because the automation of synthetic operations improves both the reproducibility and reliability of syntheses [1–4]. Synthetic chemists frequently perform repetitive processes such as the optimization of reaction conditions, construction of compound libraries, and preparation of synthetic intermediates. These operations are very time-consuming, and do not require expert knowledge and skills. Development of automated synthetic procedures and storage of relevant digital data allow anyone to reproduce the same results anytime and anywhere using the same apparatus and reagents. As a result, synthetic chemists can spend more time on advanced and challenging problems. We previously reported automated syntheses of various bioactive compounds [5–8], including taxol, using our originally developed solution-phase automated synthesizer, ChemKonzert [9].

Protected α-amino aldehydes are versatile intermediates for the synthesis of vicinal amino alcohols and important building blocks for various bioactive natural products [10–12]. In particular, Garner’s aldehyde (**4a**) is very useful as a chiral building block [13–18]. It is sufficiently stable and its configurational rigidity allows stereoselective addition of nucleophiles to the aldehyde [19].

The most conventional synthesis of **4a** involves the protection of the amine, the carboxylic acid, and the alcohol moiety of serine, and the subsequent reduction of carboxylic acid derivatives such as ester [20–27], thioester [28], or Weinreb amide [29–30] to the aldehyde. In addition, Burke and co-workers reported an asymmetric hydroformylation of 2,2-dimethyl-2,3-dihydrooxazole for the synthesis of **4a** [31]. Although various syntheses of **4a** have been established, an automated synthesis has never been demonstrated. The automated synthesis of a versatile intermediate such as **4a** will improve the overall research efficiency of synthetic chemists. Herein, we report the first solution-phase automated synthesis of Garner’s aldehyde (**4a**) and its analogues.

## Results and Discussion

**Scheme 1:**
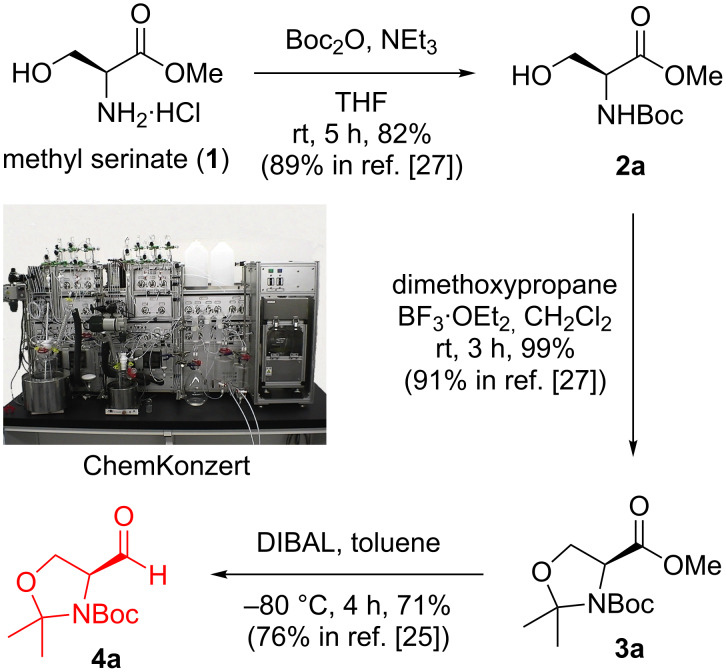
Automated synthesis of **4a**.

**Figure 1:**
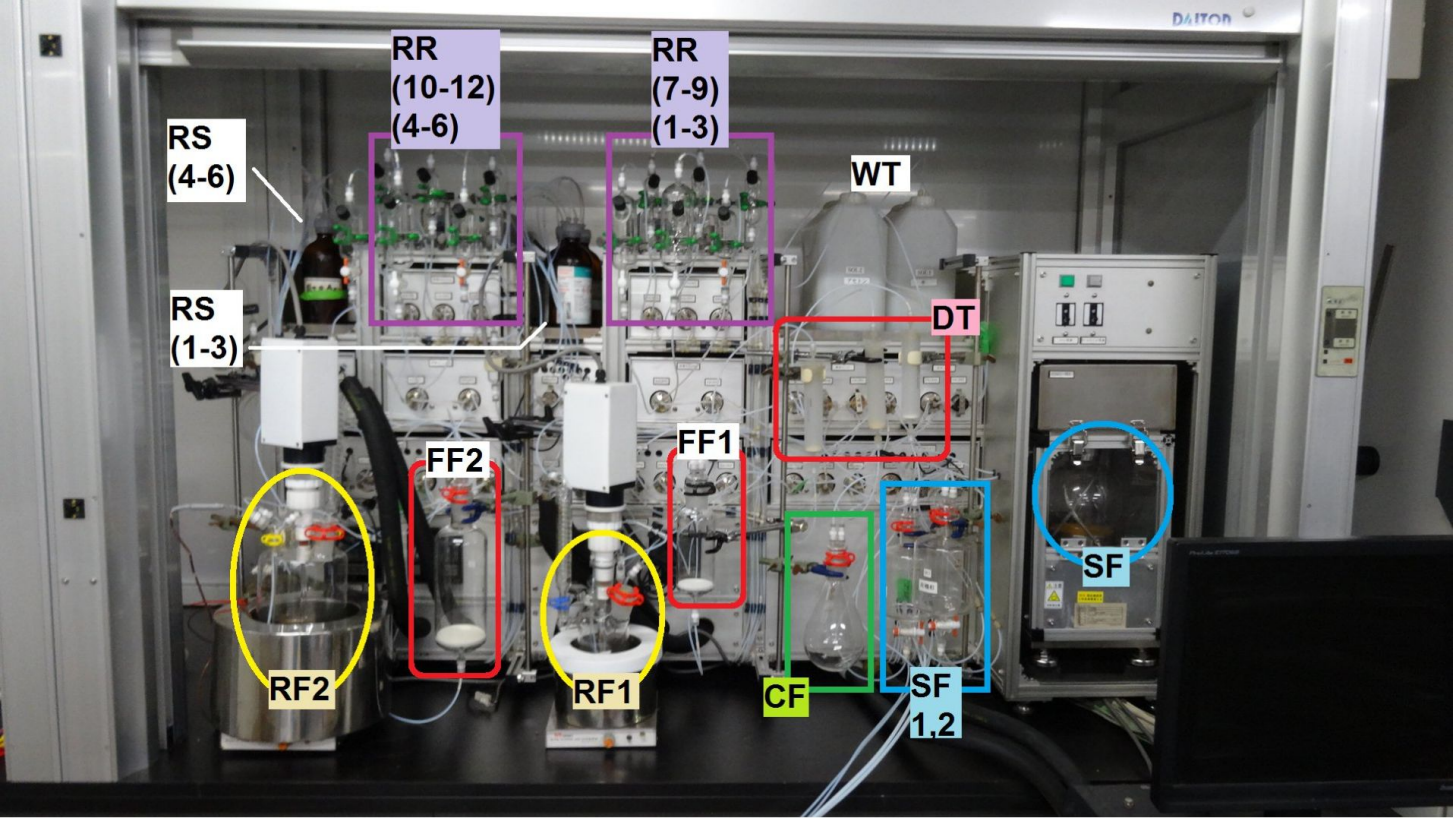
Full picture of ChemKonzert, showing two reaction vessels (RF1 and RF2), a centrifugal separator (SF, 700 mL), two receivers (SF1 and SF2, 500 mL), two glass filters (FF1 and FF2, 500 and 100 mL), 12 substrate and reagent reservoirs (RR1–RR12, 100–200 mL), six solvent and wash-solution bottles (RS1–RS6, 500 mL), three drying pads (DT1–DT3), a round-bottom flask (CF), two solvent tanks (WT1 and WT2), and a computer controller. Transfer of compounds from a server flask to a receiver flask through a Teflon tube is performed as shown below. The receiver flask is vacuumed by a diaphragm pump and N_2_ flow pushes the compound into the server flask. The flow of liquid in the tube is monitored by a photosensor that detects the difference in reflective index between gas and liquid. All the gas/liquid flows are controlled by solenoid valves and/or rotary valves. This transfer system avoids direct contacts of pumps with compounds that frequently cause mechanical troubles of pumps. Formation of emulsions during phase separation is one of the common problems for liquid-phase automated synthesizers that can perform aqueous work-up. ChemKonzert uses a centrifuge instrument to solve this problem: the emulsified mixture is transferred to the separating flask and the phases are separated by centrifugation. The separated mixture is then transferred to a receiver flask from the lower layer by passing through a flow-type electro-conductivity sensor, which detects the difference in conductivity between the organic phase and aqueous phase. When the sensor detects the boundary of the phases, the solenoid valve is changed to send the upper layer to a different receiver.

Our synthetic route is shown in Scheme 1. We planned to synthesize **4a** with various protecting groups from a commercially available amino ester through a three-step procedure utilizing the automated synthesizer, ChemKonzert (Figure 1).

Figure 1 shows the automated synthesizer ChemKonzert and its various components. An automated synthesis of **4a** was examined utilizing ChemKonzert (Scheme 1). It is important to examine and check the reaction conditions manually before performing the automated synthesis. Therefore, we optimized the reaction time and the work-up method was modified. We started with the Boc protection of methyl L-serinate hydrochloride (**1**). The computer controlling the automated synthesizer was programmed with a specific procedure. The substrate, reagents, solvents, and wash solutions were added to the reaction vessel (RF1), reagent reservoir (RR1), solvent bottles (RS1–3), and wash solution bottles (RS4–6), respectively. A solution of methyl L-serinate hydrochloride in THF was stirred at 25 °C in RF1, to which a solution of triethylamine in THF and Boc_2_O in THF was added. Originally, the respective solutions were loaded in the reagent reservoirs (RR1 and RR3). After stirring at 25 °C for 5 h, the reaction mixture was diluted with ethyl acetate from RS1 and was quenched by adding 1 M HCl from RR2. The reaction mixture was then transferred to the centrifugal separator (SF). After centrifugation, the two resulting phases were separated; their electroconductivities measured with a sensor and transferred to two receivers (SF1 and SF2). The aqueous phase in SF1 was returned to RF1. Ethyl acetate, from RS1, was added, and the mixture was stirred for 3 min and then transferred to SF. After performing the extraction, the combined organic mixture in the receiver (SF2) was washed with 10% aqueous NaCl solution from RS3. The organic layer was separated in SF, transferred to SF2, subsequently passed through a plug of anhydrous Na_2_SO_4_ (DT1) and collected in a round-bottom flask (CF1). The collected solution was manually concentrated in vacuo. The obtained residue was purified manually using silica gel column chromatography. Carbamate **2a** was obtained in 82% yield.

Acetal formation was also demonstrated using ChemKonzert. A solution of substrate **2a** in dichloromethane was stirred at 25 °C in the reaction vessel (RF1), to which a solution of 2,2-dimethoxypropane in dichloromethane and a solution of boron trifluoride·ethyl ether complex in dichloromethane were added. Originally, the respective solutions were loaded into the reagent reservoirs (RR1 and RR3). After stirring at 25 °C for 3 h, the reaction was quenched by adding 50% aqueous NaOH solution. When the NaOH solution was added to RF1, the yield of the target compound decreased because of the undesired hydrolysis of the acetonide. Therefore, the reaction mixture was transferred to the centrifugal separator (SF), NaOH solution was added to RF1 and the reaction mixture in SF was added to the NaOH solution in RF1. This reverse addition improved the yield. The subsequent automated aqueous work-up, manual concentration, and silica gel column chromatography afforded acetonide **3a** in 99% yield.

DIBAL reduction was also achieved using ChemKonzert. The amount of Rochelle salt required to diminish the aluminum salt generated from DIBAL was optimized in manual operation preliminarily. A solution of the substrate in toluene was stirred at −80 °C in the reaction vessel (RF1). A solution of DIBAL in toluene, originally loaded into the reagent reservoir (RR1), was added to RF1. After further stirring at −80 °C for 4 h, the reaction was quenched by adding saturated aqueous Rochelle salt solution at 25 °C from the solvent bottle RS2. The subsequent automated aqueous work-up, manual concentration, and silica gel column chromatography afforded **4a** in 71% yield. The observed yields of the automated syntheses were similar to those obtained from the corresponding reported manual syntheses (see Scheme 1).

Garner’s aldehyde analogues containing a Fmoc [32] or Cbz [33–35] group were synthesized using the established procedure. Protection of the amino group in methyl serinate using Fmoc–OSu or CbzCl afforded the corresponding carbamates in good yields (Table 1, experimental details, see Supporting Information File 1). Acetal formation and reduction were performed by the developed procedure in ChemKonzert (Table 1).

**Table 1:** Automated synthesis of **4b** and **4c**.

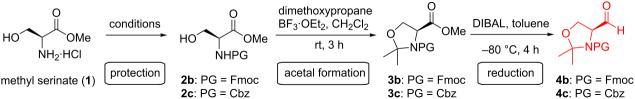			

PG	Protection	Acetal formation	Reduction

Fmoc	Fmoc–OSu, NaHCO_3_, dioxane, H_2_O, rt, 5 h, 97%	87%	22%
Cbz	CbzCl, NaHCO_3_, dioxane, H_2_O, rt, 5 h, 92%	80%	31%

The Garner’s aldehydes containing an Fmoc or Cbz protecting group (PG) could be synthesized from the corresponding methyl ester; however, lower yields were obtained for the DIBAL reduction, probably due to the DIBAL-mediated removal of the carbamates [32].

## Conclusion

In conclusion, the first solution-phase automated synthesis of **4a** (Boc protection) was demonstrated utilizing our originally developed automated synthesizer, ChemKonzert. The observed yields were comparable to those of the corresponding reported manual syntheses. In addition, **4b** and **4c** (Fmoc and Cbz protection) were also synthesized automatically according to the established procedure. Garner’s aldehyde (**4a**) and its analogues are very important versatile intermediates. The automated synthesis of **4a** can be applied to the synthesis of various useful compounds containing a vicinal amino alcohol moiety.

## Supporting Information

File 1 Synthetic procedures and ^1^H NMR spectral data of compounds **2a**–**c**, **3a**–**c**, and **4a**–**c**.
