# Supplementary material for: Solution-phase automated synthesis of an α-amino aldehyde as a versatile intermediate
Source: Beilstein J Org Chem. 2017 Jan 17;13:106–10. doi: 10.3762/bjoc.13.13 (PMC5301908; doi:10.3762/bjoc.13.13)
Supplement: File 1 — Synthetic procedures and 1H NMR spectral data of compounds 2a–c, 3a–c, and 4a–c. [file Beilstein_J_Org_Chem-13-106-s001.pdf]

Supporting Information

for

Solution-phase automated synthesis of an α-amino aldehyde as a versatile intermediate

Hisashi Masui[1], Sae Yosugi[1], Shinichiro Fuse[2] and Takashi Takahashi*[1]

Address: [1]Yokohama University of Pharmacy, 601 Matano-cho, Totsuka-ku, Yokohama 245-0066, Japan, and [2]Laboratory for Chemistry and Life Science, Institute of Innovative Research, Tokyo Institute of Technology, 4259 Nagatsuta-cho, Midori-ku, Yokohama 226-8503, Japan

Email: Takashi Takahashi - ttak@hamayaku.ac.jp

* Corresponding author

Synthetic procedures and ^{1}H NMR spectral data of compounds 2a–c, 3a–c, and 4a–c

Experimental

General

NMR spectra were recorded on a JEOL Model ECA-500 instrument. Chemical shifts are reported in parts per million (ppm) relative to the signal for the internal standard tetramethylsilane (0.0 ppm) or the solvent $CDCl_3$ (7.26 ppm, ^1H NMR; or 77.1 ppm, ^{13}C NMR) peaks. Data for ^1H NMR spectra are reported as follows: chemical shift (δ ppm), multiplicity, coupling constant (Hz), and integration. ^{13}C NMR spectrum data are reported as follows: chemical shift (δ ppm), and where applicable, multiplicity and coupling constants. Multiplicities are reported using the following abbreviations: s, singlet; d, doublet; t, triplet; q, quartet; sp, septet; m, multiplet; br, broad; and J, coupling constants in hertz. Only the strongest and/or structurally relevant IR peaks are reported (cm^{-1}). All reactions were monitored by thin-layer chromatography performed using 0.2 mm E. Merck silica gel plate (60F-254). The reactants and products were visualized using UV light (254 nm), or by heating after treatment with *p*-anisaldehyde solution, ceric sulfate solution, ninhydrin solution, or 10% ethanoic phosphomolybdic acid. Column chromatography separations were performed using silica gel (Merck).

Methyl (*tert*-butoxycarbonyl)-L-serinate (2a)

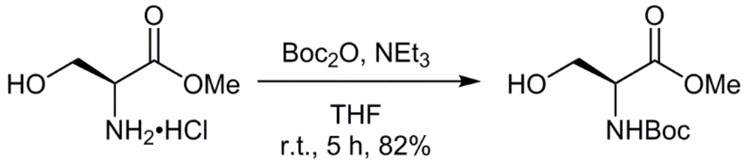

START

RR2-RF1

STIR-RF1

RR3-RF1

RR1-RF1

RR4-RR1-RF1

WAIT(Min) (Wait 300 min)

RS2-RF1

WAIT(Min) (Wait 3 min)

STIROFF-RF1

RS1-RF1

SEP-RF1 (UP)

SF1-RF1

RS1-RF1

SEP-RF1(UP)

SF2-SF

RS3-RF1

SEP-RF1(UP)

SF2-DT1-CF1

END

The reagents, washings and solvents were placed in each reservoirs and bottles as described below.

RF1 methyl L-serinate hydrochloride (2.00 g, 12.9 mmol)

RR1 Boc_2O (3.38 g, 15.5 mmol) in 11.0 mL of THF

RR2 THF (50.0 mL)

RR3 Et_3N (5.36 mL, 38.7 mmol)

RR4 THF 5.00 mL

RS1 EtOAc 120 mL

RS2 HCl (1 M aqueous) 60.0 mL

RS3 NaCl (10% aqueous) 80.0 mL

DT1 Na_2SO_4

To a solution of methyl L-serinate hydrochloride (**1**, 2.00 g, 12.9 mmol, 1.00 equiv., RF1) in THF (50.0 mL, RR2) were added Et_3N (5.36 mL, 38.7 mmol, 3.00 equiv, RR3) and Boc_2O (3.38 g, 15.5 mmol, 1.20 equiv, RR1) in THF (11.0 mL, RR1) diluted with THF (5.00 mL, RR4) and transferred to RF1 at 25 °C. After being stirred at the same temperature for 5 h, the reaction mixture (RF1) was quenched with 1 M HCl aq. (RS2) and the aqueous layer was extracted with two portions of ethyl acetate (RS1). The combined organic layer was washed with 10% NaCl aq. (RS3), dried over Na_2SO_4 (DT1), filtered, and concentrated in vacuo. The residue was purified by column chromatography on silica gel (50% ethyl acetate in hexane) to give methyl (*tert*-butoxycarbonyl)-L-serinate (2.31 g, 10.5 mmol, 82%) as a colorless oil.

^{1}H NMR (500 MHz, $CDCl_3$) δ 5.43 (s, 1H), 4.39 (s, 1H), 3.98 (dd, *J* = 4.0 Hz, 11.5 Hz, 1H), 3.93 (dd, *J* = 3.5 Hz, 11.5 Hz, 1H), 3.79 (s, 3H), 1.45 (s, 9H).

3-(*tert*-Butyl) 4-methyl (*S*)-2,2-dimethyloxazolidine-3,4-dicarboxylate (3a)

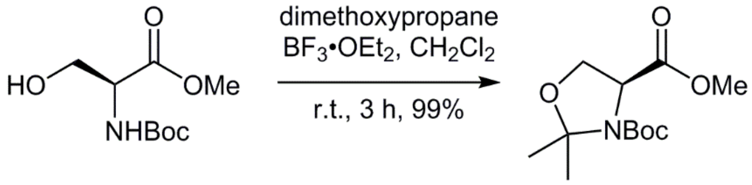

START

STIR-RF1

RR1-RF1

RR4-RR1-RF1

RR3-RF1

RR6-RR3-RF1

WAIT(Min) (Wait 360 min)

STIROFF-RF1

RF1-SF

SF-SF2

RR2-RF1

SF2-RF1

STIR-RF1

WAIT(Min) (Wait 5 min)

STIROFF-RF1

RS1-RF1

SEP-RF1(UP)

SF1-RF1

RS1-RF1

SEP-RF1(UP)

SF2-SF

RS3-RF1

SEP-RF1(UP)

SF2-DT2-CF1

END

The reagents, washings and solvents were placed in each reservoirs and bottles as described below.

RF1 methyl (*tert*-butoxycarbonyl)-L-serinate (**2a**, 1.94 g, 8.85 mmol) in 20.0 mL of CH_2Cl_2

RR1 2,2-dimethoxypropane (3.25 mL, 26.6 mmol) in 15.0 mL of CH_2Cl_2

RR2 NaOH (10% aqueous) 60.0 mL

RR3 $BF_3{\cdot}OEt_2$ (0.0556 mL, 0.443 mmol) in 15.0 mL of CH_2Cl_2

RR4 CH_2Cl_2 10.0 mL

RR6 CH_2Cl_2 10.0 mL

RS1 EtOAc 160 mL

RS3 NaCl (10% aqueous) 80.0 mL

DT2 Na_2SO_4

To a solution of methyl (*tert*-butoxycarbonyl)-L-serinate (**2a**, 1.94 g, 8.85 mmol, 1.00 equiv, RF1) in CH_2Cl_2 (20.0 mL, RF1) were added 2,2-dimethoxylpropane (3.25 mL, 26.6 mmol, 3.00 equiv, RR1) in CH_2Cl_2 (15.0 mL, RR1) diluted with CH_2Cl_2 (10.0 mL, RR4) and boron trifluoride·ethyl ether complex (0.0553 mL, 0.443 mmol, 0.500 equiv, RR3) in CH_2Cl_2 (15.0 mL, RR3) diluted with CH_2Cl_2 (10.0 mL, RR6) and transferred to RF1 at 25 °C. After being stirred at the same

temperature for 3 h, the reaction mixture (RF1) was quenched with saturated NaOH aq. (RR2) and the aqueous layer was extracted with two portions of ethyl acetate (RS1). The combined organic layer was washed with 10% NaCl aq. (RS3), dried over Na_2SO_4 (DT2), filtered, and concentrated in vacuo. The residue was purified by column chromatography on silica gel (16% ethyl acetate in hexane) to give 3-(*tert*-butyl) 4-methyl (*S*)-2,2-dimethyloxazolidine-3,4-dicarboxylate (2.28 g, 8.79 mmol, 99%) as a colorless oil.

^{1}H NMR (500 MHz, $CDCl_3$) δ 4.49 (dd, *J* = 2.5 Hz, 7.0 Hz, 1H), 4.38 (dd, *J*=3.0 Hz, 7.0 Hz, 1H), 4.16-4.12 (m, 1H), 4.04-4.02 (m, 1H), 3.75 (s, 3H), 1.67-1.41 (m, 15H).

tert-Butyl (_R_)-4-(hydroxymethyl)-2,2-dimethyloxazolidine-3-carboxylate (4a)

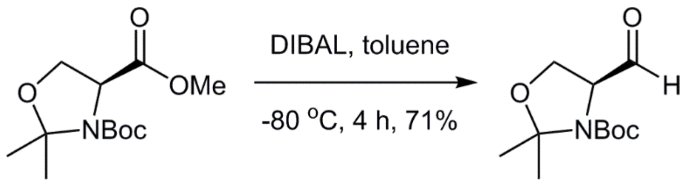

START

STIR-RF1

RR2-RF1

WAIT(Min) (Wait 240 min)

RS2-RF1

RS1-RF1

WAIT(Min) (Wait 60 min)

STIROFF-RF1

SEP-RF1(UP)

SF1-RF1

RS1-RF1

SEP-RF1(UP)

SF2-SF

RS3-RF1

SEP-RF1(UP)

SF2-DT1-CF1

END

The reagents, washings and solvents were placed in each reservoirs and bottles as described below.

RF1 3-(*tert*-butyl) 4-methyl (*S*)-2,2-dimethyloxazolidine-3,4-dicarboxylate (1.00 g, 3.86 mmol, 1.00 equiv, RF1) in 50.0 mL of toluene

RR2 Diisobutylaluminum Hydride (7.72 mL, 7.72 mmol, 2.00 equiv)

RS1 EtOAc 160 mL

RS2 Rochelle salt (10% aqueous) 60.0 mL

RS3 NaCl (10% aqueous) 80.0 mL

DT1 Na_2SO_4

To a solution of 3-(*tert*-butyl) 4-methyl (*S*)-2,2-dimethyloxazolidine-3,4-dicarboxylate (1.00 g, 3.86 mmol, 1.00 equiv, RF1) in toluene (50.0 mL, RF1) were added DIBAL (7.72 mL, 7.72 mmol, 2.00 equiv, RR2) and transferred to RF1 at −80 °C. After being stirred at the same temperature for 4 h, the reaction mixture was quenched with saturated aqueous Rochelle salt and the aqueous

layer (RS2) at 25 °C. After being stirred at the same temperature for 1 h, the reaction mixture was extracted with two portions of ethyl acetate (RS1). The combined organic layer was washed with 10% NaCl aq. (RS3), dried over Na_2SO_4 (DT1), filtered, and concentrated in vacuo. The residue was purified by column chromatography on silica gel (25% ethyl acetate in hexane) to give *tert*-butyl (*R*)-4-(hydroxymethyl)-2,2-dimethyloxazolidine-3-carboxylate (0.603 g, 2.63 mmol, 71%) as a colorless oil.

^{1}H NMR (500 MHz, $CDCl_3$) δ 9.61-9.54 (m, 1H), 4.35-4.18 (m, 1H), 4.12-4.02 (m, 2H), 1.65-1.41 (m, 15H).

Methyl (((9*H*-fluoren-9-yl)methoxy)carbonyl)-L-serinate (2b)

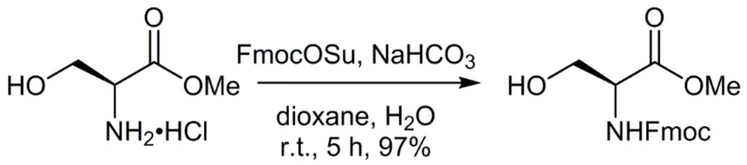

START

RR2-RF1

STIR-RF1

RR3-RF1

RR1-RF1

RR4-RR1-RF1

WAIT(Min) (Wait 300 min)

RS2-RF1

WAIT(Min) (Wait 3 min)

STIROFF-RF1

RS1-RF1

SEP-RF1 (UP)

SF1-RF1

RS1-RF1

SEP-RF1(UP)

SF2-SF

RS3-RF1

SEP-RF1(UP)

SF2-DT2-CF1

END

The reagents, washings and solvents were placed in each reservoirs and bottles as described below.

RF1	methyl L-serinate hydrochloride (1.00 g, 6.31 mmol)
RR1	Fmoc-OSu (2.55 g, 7.57 mmol) in 15.0 mL of dioxane
RR2	dioxane (5.00 mL)
RR3	$NaHCO_3$ (1.59 g, 18.9 mmol) in 25.2 mL of H_2O
RR4	dioxane 4.20 mL
RS1	EtOAc 120 mL
RS2	HCl (1 M aqueous) 60.0 mL
RS3	NaCl (10% aqueous) 80.0 mL
DT2	Na_2SO_4

To a solution of methyl L-serinate hydrochloride (1.00 g, 6.31 mmol, 1.00 equiv., RF1) in dioxane (5.00 mL, RR2) were added $NaHCO_3$ (1.59 g, 18.9 mmol, 3.00

equiv, RR3) in H_2O (25.2 mL, RR3) and Fmoc-OSu (2.55 g, 7.57 mmol, 1.20 equiv, RR1) in dioxane (15.0 mL, RR1) diluted with dioxane (4.20 mL, RR4) and transferred to RF1 at 25 °C. After being stirred at the same temperature for 5 h, the reaction mixture (RF1) was quenched with 1 M HCl aq. (RS2) and the aqueous layer was extracted with two portions of ethyl acetate (RS1). The combined organic layer was washed with 10% NaCl aq. (RS3), dried over Na_2SO_4 (DT2), filtered, and concentrated in vacuo. The residue was purified by column chromatography on silica gel (50% ethyl acetate in hexane) to give methyl (((9*H*-fluoren-9-yl)methoxy)carbonyl)-L-serinate (2.08 g, 6.09 mmol, 97%) as a colorless oil.

^{1}H NMR (500 MHz, $CDCl_3$) δ 7.78-7.61 (m, 4H), 7.43-7.31 (m, 4H), 5.67 (d, *J* = 7.0 Hz, 1H), 4.44-4.43 (m, 3H), 4.24 (t, *J* = 6.5 Hz, 1H), 4.02-3.94 (m, 2H), 3.80 (s, 3H), 2.05 (t, *J* = 6.0 Hz, 1 H).

3-((9*H*-Fluoren-9-yl)methyl) 4-methyl (*S*)-2,2-dimethyloxazolidine-3,4-dicarboxylate (3b)

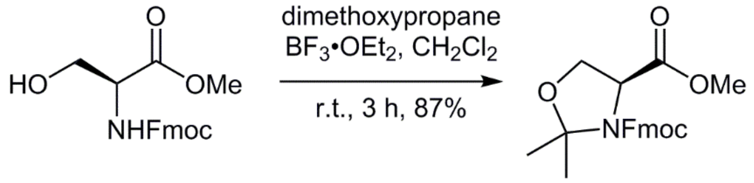

START

STIR-RF1

RR1-RF1

RR4-RR1-RF1

RR3-RF1

RR6-RR3-RF1

WAIT(Min) (Wait 360 min)

STIROFF-RF1

RF1-SF

SF-SF2

RR2-RF1

SF2-RF1

STIR-RF1

WAIT(Min) (Wait 5 min)

STIROFF-RF1

RS1-RF1

SEP-RF1(UP)

SF1-RF1

RS1-RF1

SEP-RF1(UP)

SF2-SF

RS3-RF1

SEP-RF1(UP)

SF2-DT2-CF1

END

The reagents, washings and solvents were placed in each reservoirs and bottles as described below.

RF1 methyl (((9*H*-fluoren-9-yl)methoxy)carbonyl)-L-serinate (1.00 g, 2.93 mmol) in 15.6 mL of CH_2Cl_2

RR1 2,2-dimethoxypropane (1.08 mL, 8.79 mmol) in 5.00 mL of CH_2Cl_2

RR2 NaOH (10% aqueous) 40.0 mL

RR3 $BF_3 \cdot OEt_2$ (0.0368 mL, 0.293 mmol) in 15.0 mL of CH_2Cl_2

RR4 CH_2Cl_2 5.00 mL

RR6 CH_2Cl_2 5.00 mL

RS1 EtOAc 160 mL

RS3 NaCl (10% aqueous) 80.0 mL

DT2 Na_2SO_4

To a solution of methyl (((9*H*-fluoren-9-yl)methoxy)carbonyl)-L-serinate (**2b**, 1.00 g, 2.93 mmol, 1.00 equiv, RF1) in CH_2Cl_2 (15.6 mL, RF1) were added 2,2-dimethoxylpropane (1.08 mL, 8.79 mmol, 3.00 equiv, RR1) in CH_2Cl_2 (5.00 mL, RR1) diluted with CH_2Cl_2 (5.00 mL, RR4) and boron trifluoride·ethyl ether complex (0.0368 mL, 0.293 mmol, 0.100 equiv., RR3) in CH_2Cl_2 (15.0 mL, RR3) diluted with CH_2Cl_2 (5.00 mL,RR6) and transferred to RF1 at 25 °C. After being stirred at the same temperature for 3 h, the reaction mixture (RF1) was quenched with saturated NaOH aq. (RR2) and the aqueous layer was extracted with two portions of ethyl acetate (RS1). The combined organic layer was washed with 10% NaCl aq. (RS3), dried over Na_2SO_4 (DT2), filtered, and concentrated in vacuo. The residue was purified by column chromatography on silica gel (25% ethyl acetate in hexane) to give 3-(*tert*-butyl) 3-((9*H*-fluoren-9-yl)methyl) 4-methyl (*S*)-2,2-dimethyloxazolidine-3,4-dicarboxylate (0.977 g, 2.56 mmol, 87%) as a colorless oil.

^{1}H NMR (500 MHz, $CDCl_3$) δ 7.74-7.53 (m, 4H), 7.39-7.30 (m, 4H), 4.72 (t, *J* = 4.5 Hz, 1H), 4.47-4.07 (m, 5H), 3.65 (s, 3H), 1.73 (s, 3H), 1.53 (s, 3H).

(9*H*-Fluoren-9-yl)methyl (*S*)-4-formyl-2,2-dimethyloxazolidine-3-carboxylate (4b)

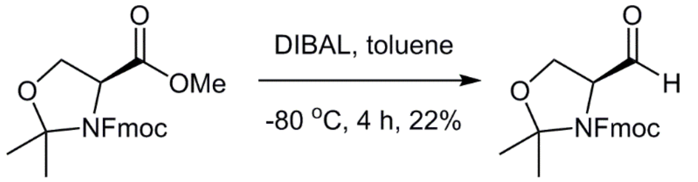

START

STIR-RF1

RR2-RF1

WAIT(Min) (Wait 240 min)

RS2-RF1

RS1-RF1

WAIT(Min) (Wait 60 min)

STIROFF-RF1

SEP-RF1(UP)

SF1-RF1

RS1-RF1

SEP-RF1(UP)

SF2-SF

RS3-RF1

SEP-RF1(UP)

SF2-DT1-CF1

END

The reagents, washings and solvents were placed in each reservoirs and bottles as described below.

RF1 3-((9*H*-fluoren-9-yl)methyl) 4-methyl (*S*)-2,2-dimethyloxazolidine-3,4-dicarboxylate (1.00 g, 2.62 mmol, 1.00 equiv., RF1) in 52.4 mL of toluene

RR2 Diisobutylaluminum hydride (5.24 mL, 5.24 mmol, 2.00 equiv.)

RS1 EtOAc 160 mL

RS2 Rochelle salt (10% aqueous) 60.0 mL

RS3 NaCl (10% aqueous) 80.0 mL

DT1 Na_2SO_4

To a solution of 3-((9*H*-fluoren-9-yl)methyl) 4-methyl (*S*)-2,2-dimethyloxazolidine-3,4-dicarboxylate (**3b**, 1.00 g, 2.62 mmol, 1.00 equiv, RF1) in toluene (52.4 mL, RF1) were added DIBAL (5.24 mL, 5.24 mmol, 2.00 equiv, RR2) and transferred to RF1 at −80 °C. After being stirred at the same temperature for 4 h, the reaction mixture was quenched with saturated aqueous Rochelle salt and the aqueous layer (RS2) at 25 °C. After being stirred at the same temperature for 1 h, the reaction mixture was extracted with two portions of ethyl acetate (RS1). The combined organic layer was washed with 10% NaCl aq. (RS3), dried over Na_2SO_4 (DT1), filtered, and concentrated in vacuo. The residue was purified by column chromatography on silica gel (25% ethyl acetate in hexane) to give (9*H*-fluoren-9-yl)methyl (*S*)-4-formyl-2,2-dimethyloxazolidine-3-carboxylate (0.206 g, 0.586 mmol, 22%) as a colorless oil.

^{1}H NMR (500 MHz, $CDCl_3$) δ 9.14 (s, 1H) 7.71-7.51 (m, 4H), 7.41-7.31 (m, 4H), 4.80 (m, 1H), 4.62-3.89 (m, 5H), 1.60 (s, 3H), 1.49 (s, 3H).

Methyl ((benzyloxy)carbonyl)-L-serinate (2c)

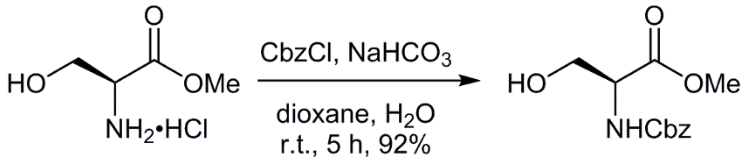

START

RR2-RF1

STIR-RF1

RR3-RF1

RR1-RF1

RR4-RR1-RF1

WAIT(Min) (Wait 300 min)

RS2-RF1

WAIT(Min) (Wait 3 min)

STIROFF-RF1

RS1-RF1

SEP-RF1 (UP)

SF1-RF1

RS1-RF1

SEP-RF1(UP)

SF2-SF

RS3-RF1

SEP-RF1(UP)

SF2-DT2-CF1

END

The reagents, washings and solvents were placed in each reservoirs and bottles as described below.

RF1 methyl L-serinate hydrochloride (1.00 g, 6.43 mmol)

RR1 Cbz-Cl (1.10 mL, 7.72 mmol) in 15.0 mL of dioxane

RR2 dioxane (5.00 mL)

RR3 $NaHCO_3$ (1.62 g, 19.3 mmol) in 25.7 mL of H_2O

RR4 dioxane 4.20 mL

RS1 EtOAc 120 mL

RS2 HCl (1 M aqueous) 60.0 mL

RS3 NaCl (10% aqueous) 80.0 mL

DT2 Na_2SO_4

To a solution of methyl L-serinate hydrochloride (**1**, 1.00 g, 6.43 mmol, 1.00 equiv, RF1) in dioxane (5.00 mL, RR2) were added $NaHCO_3$ (1.62 g, 19.3 mmol, 3.00 equiv, RR3) in H_2O (25.7 mL, RR3) and Cbz-Cl (1.10 mL, 7.72 mmol, 1.20 equiv, RR1) in dioxane (15.0 mL, RR1) diluted with dioxane (4.20 mL, RR4) and transferred to RF1 at 25 °C. After being stirred at the same temperature for 5 h, the reaction mixture (RF1) was quenched with 1 M HCl aq. (RS2) and the aqueous layer was extracted with two portions of ethyl acetate (RS1). The combined organic layer was washed with 10% NaCl aq. (RS3), dried over Na_2SO_4 (DT2), filtered, and concentrated in vacuo. The residue was

purified by column chromatography on silica gel (50% ethyl acetate in hexane) to give methyl ((benzyloxy)carbonyl)-L-serinate (1.50 g, 5.92 mmol, 92%) as a colorless oil.

^{1}H NMR (500 MHz, $CDCl_3$) δ 7.37-7.31 (m, 5H), 5.74 (s, 1H), 5.12 (s, 2H), 4.45 (t, J = 7.5 Hz, 1H), 4.01-3.91 (m, 2H), 3.78 (s, 3H), 2.34 (s, 1H).

3-benzyl 4-methyl (*S*)-2,2-dimethyloxazolidine-3,4-dicarboxylate (3c)

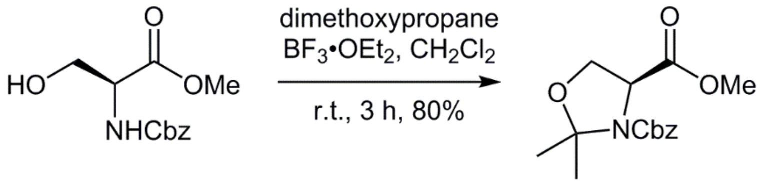

START

STIR-RF1

RR1-RF1

RR4-RR1-RF1

RR3-RF1

RR6-RR3-RF1

WAIT(Min) (Wait 360 min)

STIROFF-RF1

RF1-SF

SF-SF2

RR2-RF1

SF2-RF1

STIR-RF1

WAIT(Min) (Wait 5 min)

STIROFF-RF1

RS1-RF1

SEP-RF1(UP)

SF1-RF1

RS1-RF1

SEP-RF1(UP)

SF2-SF

RS3-RF1

SEP-RF1(UP)

SF2-DT2-CF1

END

The reagents, washings and solvents were placed in each reservoirs and bottles as described below.

RF1 methyl ((benzyloxy)carbonyl)-L-serinate (7.00 g, 27.6 mmol) in 30.0 mL of CH_2Cl_2

RR1 2,2-dimethoxypropane (10.1 mL, 82.8 mmol) in 10.0 mL of CH_2Cl_2

RR2 NaOH (10% aqueous) 40.0 mL

RR3 $BF_3 \cdot OEt_2$ (0.347 mL, 2.76 mmol) in 5.00 mL of CH_2Cl_2

RR4 CH_2Cl_2 5.00 mL

RR6 CH_2Cl_2 5.00 mL

RS1 EtOAc 160 mL

RS3 NaCl (10% aqueous) 80.0 mL

DT2 Na_2SO_4

To a solution of methyl ((benzyloxy)carbonyl)-L-serinate (7.00 g, 27.6 mmol, 1.00 equiv, RF1) in CH_2Cl_2 (30.0 mL, RF1) were added 2,2-dimethoxypropane (10.1 mL, 82.8 mmol, 3.00 equiv, RR1) in CH_2Cl_2 (10.0 mL, RR1) diluted with CH_2Cl_2 (5.00 mL, RR4) and boron trifluoride·ethyl ether complex (0.347 mL, 2.76 mmol, 0.100 equiv, RR3) in CH_2Cl_2 (5.00 mL, RR3) diluted with CH_2Cl_2 (5.00 mL, RR6) and transferred to RF1 at 25 °C. After being stirred at the same temperature for 3 h, the reaction mixture (RF1) was quenched with saturated NaOH aq. (RR2) and the aqueous layer was extracted with two portions of ethyl acetate (RS1). The combined organic layer was washed with 10% NaCl aq. (RS3), dried over Na_2SO_4 (DT2), filtered, and concentrated in vacuo. The residue was purified by column chromatography on silica gel (16% ethyl acetate in hexane) to give 3-benzyl 4-methyl (*S*)-2,2-dimethyloxazolidine-3,4-dicarboxylate (6.44 g, 22.0 mmol, 80%) as a colorless oil.

^{1}H NMR (500 MHz, $CDCl_3$) δ 7.37-7.30 (m, 5H), 5.23-5.03 (m, 2H), 4.55-4.47 (m, 2H), 4.18-4.08 (m, 1H), 3.64 (s, 3H), 1.71 (s, 3H), 1.56 (s, 3H).

Benzyl (*S*)-4-formyl-2,2-dimethyloxazolidine-3-carboxylate (4c)

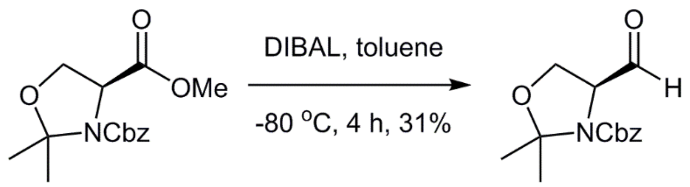

START

STIR-RF1

RR2-RF1

WAIT(Min) (Wait 240 min)

RS2-RF1

RS1-RF1

WAIT(Min) (Wait 60 min)

STIROFF-RF1

SEP-RF1(UP)

SF1-RF1

RS1-RF1

SEP-RF1(UP)

SF2-SF

RS3-RF1

SEP-RF1(UP)

SF2-DT1-CF1

END

The reagents, washings and solvents were placed in each reservoirs and bottles as described below.

RF1 3-benzyl 4-methyl (*S*)-2,2-dimethyloxazolidine-3,4-dicarboxylate (1.00 g, 3.41 mmol, 1.00 equiv., RF1) in 40.0 mL of toluene

RR2 Diisobutylaluminum Hydride (6.82 mL, 6.82 mmol, 2.00 equiv.)

RS1 EtOAc 160 mL

RS2 Rochelle salt (10% aqueous) 60.0 mL

RS3 NaCl (10% aqueous) 80.0 mL

DT1 Na_2SO_4

To a solution of 3-benzyl 4-methyl (*S*)-2,2-dimethyloxazolidine-3,4-dicarboxylate (**3c**, 1.00 g, 3.41 mmol, 1.00 equiv, RF1) in toluene (40.0 mL, RF1) were added DIBAL (6.82 mL, 6.82 mmol, 2.00 equiv, RR2) and transferred to RF1 at −80 °C. After being stirred at the same temperature for 4 h, the reaction mixture was quenched with saturated aqueous Rochelle salt and the aqueous layer (RS2) at 25 °C. After being stirred at the same temperature for 1 h, the reaction mixture was extracted with two portions of ethyl acetate (RS1). The combined organic layer was washed with 10% NaCl aq. (RS3), dried over Na_2SO_4 (DT1), filtered, and concentrated in vacuo. The residue was purified by column chromatography on silica gel (25% ethyl acetate in hexane) to give benzyl (*S*)-4-formyl-2,2-dimethyloxazolidine-3-carboxylate (0.276 g, 1.05 mmol, 31%) as a colorless oil.

^{1}H NMR (500 MHz, $CDCl_3$) δ 9.53 (s, 1H) 7.36-7.28 (m, 5H), 5.22-5.01 (m, 2H), 4.41-4.31 (m, 2H), 4.12-4.02 (m, 1H), 1.66 (s, 3H), 1.56 (s, 3H).
